# Developmental dyscalculia is related to visuo-spatial memory and inhibition impairment^☆^

**DOI:** 10.1016/j.cortex.2013.06.007

**Published:** 2013-11

**Authors:** Denes Szucs, Amy Devine, Fruzsina Soltesz, Alison Nobes, Florence Gabriel

**Affiliations:** a Department of Psychology, Centre for Neuroscience in Education, University of Cambridge, Cambridge, United Kingdom; b Department of Psychiatry, University of Cambridge, United Kingdom

**Keywords:** Developmental disorders, Intraparietal sulcus (IPS), Developmental learning disability, Mathematical difficulty, Number sense

## Abstract

Developmental dyscalculia is thought to be a specific impairment of mathematics ability. Currently dominant cognitive neuroscience theories of developmental dyscalculia suggest that it originates from the impairment of the magnitude representation of the human brain, residing in the intraparietal sulcus, or from impaired connections between number symbols and the magnitude representation. However, behavioral research offers several alternative theories for developmental dyscalculia and neuro-imaging also suggests that impairments in developmental dyscalculia may be linked to disruptions of other functions of the intraparietal sulcus than the magnitude representation. Strikingly, the magnitude representation theory has never been explicitly contrasted with a range of alternatives in a systematic fashion. Here we have filled this gap by directly contrasting five alternative theories (magnitude representation, working memory, inhibition, attention and spatial processing) of developmental dyscalculia in 9–10-year-old primary school children. Participants were selected from a pool of 1004 children and took part in 16 tests and nine experiments. The dominant features of developmental dyscalculia are visuo-spatial working memory, visuo-spatial short-term memory and inhibitory function (interference suppression) impairment. We hypothesize that inhibition impairment is related to the disruption of central executive memory function. Potential problems of visuo-spatial processing and attentional function in developmental dyscalculia probably depend on short-term memory/working memory and inhibition impairments. The magnitude representation theory of developmental dyscalculia was not supported.

Developmental dyscalculia (DD) is a learning difficulty specific to mathematics which may affect 3–6% of the population. Pure DD (hereafter: DD) does not have apparent co-morbidity with any other developmental disorder, such as dyslexia or attention deficit hyperactivity disorder (ADHD), intelligence is normal, the only apparent weakness is in the domain of mathematics (Shalev and Gross-Tsur, 2001). The currently dominant neuroscience theory of DD assumes that DD is related to the impairment of a magnitude representation (MR) often called the approximate number system (ANS; Piazza et al., 2010) or a ‘number module’ (Landerl et al., 2004) residing in the bilateral intraparietal sulci (IPSs). This MR is thought to enable the intuitive understanding of numerical magnitude enabling number discrimination (e.g., Dehaene, 1997; Piazza et al., 2010). The MR theory of DD suggests that an impairment of the MR per se impacts on numerical skills leading to DD (Piazza et al., 2010; Landerl et al., 2004). The theory expects that non-symbolic numerosity comparison (e.g., comparing the number of items in two groups) is deficient in DD children. Another version of the MR theory assumes that the MR itself may be intact in DD but links between the MR and numerical symbols are impaired. This version expects that non-symbolic numerosity comparison is intact but symbolic numerosity comparison is deficient in DD (Rousselle and Noël, 2007; De Smedt and Gilmore, 2011). The MR theory of DD also claims support from neuro-imaging evidence because children with DD were shown to have lower gray matter density in the parietal cortex than controls in structural magnetic resonance imaging (MRI) studies (Isaacs et al., 2001; Rotzer et al., 2008; Rykhlevskaia et al., 2009) and they *sometimes* show different IPS activation relative to controls in magnitude comparison tasks in functional MRI (fMRI) studies. Strikingly, the MR theory of DD has never been systematically contrasted with various alternative theories proposed by extensive behavioral research. Here we report such a study.

The most established markers of the MR are behavioral ratio and distance effects (Moyer and Landauer, 1967) in symbolic (e.g., ‘Which is larger; 3 or 4?’) and non-symbolic (e.g., ‘Do you see more dots on the left or on the right?’) magnitude comparison tasks (ratio and distance effects refer to the fact that it is faster and less error prone to compare further away than closer quantities) and their correlates in the IPS (Pinel et al., 2001). To date five fMRI studies compared distance/ratio effects in DD and controls (Kucian et al., 2006, 2011; Price et al., 2007; Mussolin et al., 2010b; Kovas et al., 2009) and one fMRI study compared approximate calculation (performance on this is expected to rely on the MR of the IPS) in DD and controls (Davis et al., 2009). Behaviorally, only Price et al. (2007) reported a different accuracy distance effect in DD relative to controls. None of the studies reported a different reaction time (RT) distance effect in DD relative to controls. Price et al. (2007; non-symbolic comparison with no control task) and Mussolin et al. (2010b; one-digit Arabic number comparison with color comparison control task) reported weaker IPS distance effects in DD than in controls. Kucian et al. (2006; non-symbolic magnitude comparison with color comparison control task) compared activity in a greyscale comparison control task and in a magnitude comparison task but did not find any brain activity difference between DD and controls in either multiple testing corrected or uncorrected whole-brain analyses. Kovas et al. (2009; non-symbolic magnitude comparison with five ratios; with color comparison control task) reported DD versus control and numerical versus control task differences in various brain regions but not in the IPS and, in fact did not find any ratio/distance effects in the IPS. They concluded that the IPS based MR theory of DD may not stand. Kucian et al. (2011; non-symbolic magnitude comparison with no control task) observed differences between DD and controls in several brain areas but not in the parietal lobe and concluded that DD children have difficulty in response selection relative to control children. Davis et al. (2009) did not find IPS differences between DD and controls in an approximate calculation task.

In summary, evidence suggesting that abnormal IPS function is related to the MR in DD is weak. Four out of six studies returned negative fMRI findings with regard to the IPS based MR hypothesis of DD. Of the two positive studies, only one had supporting behavioral evidence (Price et al., 2007). However, this study did not use a control task, DD showed a normal RT distance effect, there was 17.7 points difference between DD and control on the Wechsler Intelligence Scale for Children (WISC) Block Design test, and memory/attention was not tested. Mussolin et al. (2010b) had a control task but did not have supporting behavioral evidence. The lack of behavioral evidence and control tasks leaves it unclear whether differences in IPS structure and perhaps function relate to numerical skill or to some other uncontrolled and untested function (Poldrack, 2006). In addition, each study tested a relatively narrow range of variables.

Purely behavioral studies arguing in favor of the MR theory used dot comparison tasks and showed that functional markers of comparison performance differed in DD and control participants (Piazza et al., 2010; Mazzocco et al., 2011; Mussolin et al., 2010a). However, none of these studies used non-numerical tasks controlling for non-numerical aspects of comparisons. Nevertheless, evidence demonstrates that both symbolic and non-symbolic comparison performance primarily reflects domain general comparison processes rather than properties of the number representation (Holloway and Ansari, 2008). Hence, the omission of a control task is a significant shortcoming and, in principle, studies without control tasks cannot draw any number-specific conclusions. In addition, the dot comparison task is inherently confounded by non-numerical parameters which cannot be controlled in each particular trial (Gebuis and Reynvoet, 2011, 2012; Szucs et al., 2013). Further, when tracking both numerical and non-numerical parameters in dot comparison tasks, event-related brain potentials (ERPs) only showed sensitivity to non-numerical parameters but not to numerical parameters (Gebuis and Reynvoet, 2012). Hence, in the dot comparison task participants' supposedly numerical judgments can rely on non-numerical parameters in each particular trial. This problem also affects fMRI studies using non-symbolic magnitude comparison. It is noteworthy that Landerl et al. (2004) is one of the most often cited studies in support of the MR theory. However, that study *merely* demonstrated that DD have *slower* magnitude comparison speed than controls which can happen for many reasons. The distance effects did not differ in DD and controls and DD *only* showed a *marginally* steeper counting range RT curve than controls (pp. 117 and 119–120). In fact, the distance effect was not significant even in controls which suggests lack of power. In an extensive follow-up study Landerl and Kolle (2009) could not detect any robust basic number processing difference between DD and controls and they concluded that they ‘did not find strong evidence that DD children process numbers qualitatively differently from children with typical arithmetic development’ (ibid., abstract).

While the MR theory of DD currently dominates neuroscience research, behavioral research identified several cognitive functions which play an important role in mathematical development and proposed several alternative theories of DD which have mostly been neglected by neuro-imaging research. First, a large volume of studies found deficient verbal and/or visuo-spatial WM function in DD (e.g., Hitch and McAuley, 1991; Passolunghi and Siegel, 2001, 2004; Keeler and Swanson, 2001; Bull et al., 2008; Swanson, 2006; Geary, 2004) and longitudinal studies confirmed that WM function is related to mathematical performance (Geary, 2011; Swanson, 2011; Passolunghi and Lanfranchi, 2012). WM serves as a limited capacity mental workspace for operands, operators, and retrieved numerical facts which have to be mobilized even during the simplest calculations (Geary, 1993; Ashcraft, 1995). Hence, its impairment can have detrimental consequences for mathematical function. Second, some studies reported spatial processing problems in DD (Rourke and Conway, 1997; Rourke, 1993) which may be related to visuo-spatial WM problems. Spatial processes can be potentially important in mathematics where explicit or implicit visualization is required, like when imagining operations along the number line or visualizing functional relationships.

Third, others found deficient inhibitory function in DD and/or a relationship between inhibitory function and mathematical development (Bull and Scerif, 2011; Bull et al., 1999; Pasolunghi et al., 1999; Passolunghi and Siegel, 2004; McKenzie et al., 2003; Espy et al., 2004; Blair and Razza, 2007; Swanson, 2011). Fourth, similar findings were reported with regard to attentional function (Swanson, 2011; Ashkenazi et al., 2009; Hannula et al., 2010). Inhibitory and attentional processes co-ordinate which items of interest receive processing and when and in what order they enter processing. This also assures that (temporarily) irrelevant potential mathematical processing events are suppressed (e.g., Barrouillet et al., 1997; Bull et al., 1999; Pasolunghi et al., 1999; Passolunghi and Siegel, 2004). Such processes are extremely important in calculations which require the continuous selection and coordination of several processing steps and items in memory. In fact, inhibitory function, attentional and working memory (WM) processes may all be intricately intertwined and form the core of so-called ‘central executive’ memory processes (Hasher and Zacks, 1988; Miyake et al., 2000).

Crucially, all of the above cognitive functions have been linked to the IPS. Hence, impairment of any of the above functions could plausibly explain IPS abnormality in DD which is routinely cited in support of the impaired MR theory of DD. IPS activity has been shown to be modulated by manipulations in WM (Culham and Kanwisher, 2001; Coull and Frith, 1998; Linden et al., 2003; Todd and Marois, 2004; Dumontheil and Klingberg, 2011), attention (Coull and Frith, 1998; Vandenberghe et al., 2012; Santangelo and Macaluso, 2013; Davranche et al., 2011), inhibitory function (Cieslik et al., 2011; Mecklinger et al., 2003) and spatial processing (Yang et al., 2011) tasks. Moreover, one study demonstrated decreased IPS function in DD children in a spatial WM task (Rotzer et al., 2009) and another study demonstrated that brain activity during a visuo-spatial WM task in the IPS predicts mathematical ability 2 years later (Dumontheil and Klingberg, 2011). Hence, IPS dysfunction in DD may well be linked to WM dysfunction. In addition, an ERP investigation of DD found that short latency (200 msec) ERPs, probably related to automatic magnitude discrimination, were similar in DD and controls but later (600 msec latency) processes indexed by the P3b wave, usually related to categorization decision, differed (Soltész et al., 2007). These findings have been confirmed by a recent study (Heine et al., 2012). Further, Soltesz et al. (2007) found that the DD and control groups differed in neuropsychological tests measuring executive functioning. Hence, it was concluded that basic number processing was intact while aspects of higher level executive memory or attention function were impaired in DD.

Overall, a serious shortcoming of the existing literature is that the MR theory has never been directly contrasted systematically with alternative theories of DD. That is, most behavioral studies focusing on memory and attention function did not use measures of the MR and most MR studies did not use a wide range of alternative measures. Here, our intention was to understand the complexity of DD by taking a very wide range of measurements. This allowed us to directly contrast the MR, WM, inhibition, attention and spatial processing theories of DD in primary school children. We matched controls for verbal and non-verbal IQ, socio-economic status and general processing speed. We used five experimental measures of the MR theory with high trial numbers. We assumed that if MR theory is correct then there should be robust differences on MR-related measures between DD and control participants on all of these tasks, especially on the non-symbolic and symbolic magnitude decision tasks which are proposed to be the most important markers of the MR. Verbal and visuo-spatial short-term memory (STM)/WM were tested by standardized measures. Inhibition performance was measured by detecting numerical and non-numerical congruency effects in four experiments and with a Stop-signal task. Sustained attention and simple RT speed were tested by visual target detection experiments. Spatial processing was measured by testing both performance scores and solution speed on a spatial symmetry task and on a mental rotation task.

## 1 Materials and methods

Methods are described in more detail in Supplementary methods. Parental consent was obtained for all phases of the study. The study received ethical approval from the Cambridge Psychology Research Ethics Committee.

### 1.1 Screening

In a first step, 1004 children were screened for DD with age-standardized United Kingdom National Curriculum-based maths and reading tests, administered to whole classes. The maths test was the Mathematics Assessment for Learning and Teaching test (MaLT; Williams, 2005), a written test containing questions covering all areas of the maths curriculum. This test allows for invigilators to read the questions to the children if required to ensure test performance reflects mathematics ability rather than reading proficiency. Reading ability was assessed using the Hodder Group Reading Test II, levels 1 and 2 (HGRT-II; Vincent and Crumpler, 2007). These multi-choice tests assess children's reading of words, sentences and passages. Characteristics of the screening sample have been described by Devine et al. (2013).

In a second step about 200 children representing the distribution of mathematics and reading scores were invited to take part in further study. A part of this sample consented and a subgroup of 115 children from the original sample took part in further screening and experimental tasks. Each child was tested for about 7–8 h duration in multiple sessions. Children were individually administered an additional standardized measure of mathematical ability [the Numerical Operations subtest of Wechsler Individual Achievement Test (WIAT-II; Wechsler, 2005)], two additional standardized measures of reading ability (WIAT-II Word Reading and Pseudoword Decoding subtests), and two IQ tests [the Raven's Colored Progressive Matrices (Raven's CPM; Raven, 2008) and a short form of the WISC – 3rd Edition (WISC-III, Wechsler, 1991)]. The WISC-III short form included the Block Design (non-verbal) and Vocabulary (verbal) subtests. This combination of subtests has the highest validity and reliability of the two-subtest forms (*r*_tt_ = .91, *r* = .86; Table L-II, Sattler, 1992). Socio-economic status was estimated from parents' education levels and occupations.

### 1.2 Participants

**Fig. 1:**
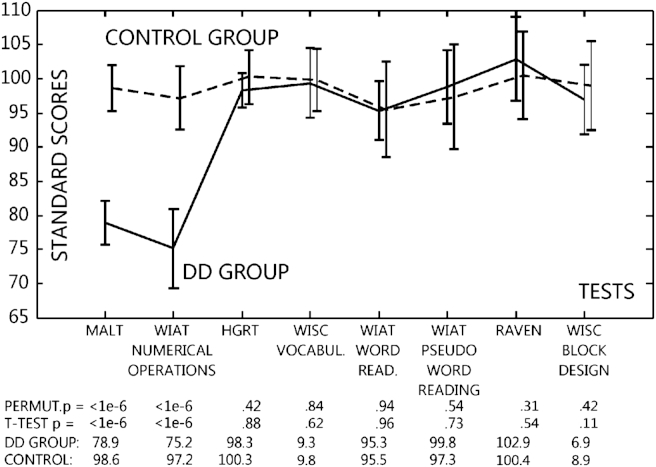
Group profiles on standardized screening tests. Group means and 95% confidence intervals are shown. Means permutation *p* and independent *t*-test *p* values are given below the *X* axis. For display purposes only the WISC Vocabulary and Block Design scores were rescaled to mean = 100 and SD = 15; analyses were done on original values which are shown numerically.

Children were defined to have DD if their mean performance on the standardized MaLT and WIAT-II UK Numerical Operations tests was worse than mean − 1SD (<16th percentile) and their performance on the HGRT-II, WISC Vocabulary, WIAT Word Reading, WIAT Pseudoword reading, Raven and WISC Block Design tests was in the mean ± 1SD range. 18 children (15.6% of the 115 children and 1.8% of the sample of 1004 children) performed worse in mathematics than the mean − 1SD criterion. Six children had both weak mathematics and reading/IQ performance (score < mean − 1SD) and were not investigated further. That is, there were 12 participants in both the DD and the Control group (DD: four girls; Control: seven girls). Criterion test profiles with standard test scores are shown in Fig. 1. Groups were perfectly matched on age (DD *vs* Control: 110 *vs* 109 months, *p* = .52), non-verbal IQ, verbal IQ and socio-economic status [parental occupation (mean and standard error (SE) for DD *vs* Controls: 4.0 ± .6 *vs* 3.7 ± .4) and parental education (4.7 ± .4 *vs* 4.9 ± .3); Mann–Whitney *U* test for both *p* > .71]. Groups differed only on the MaLT and WIAT Numerical Operations tests. It is important to point out that many studies do not match groups perfectly along variables which may affect group differences in the dependent variable and instead rely on analysis of covariance (ANCOVA) to supposedly ‘correct for’ group differences. However, this is a statistically invalid procedure and therefore an improper use of ANCOVA (see e.g., Miller and Chapman, 2001; Porter and Raudenbush, 1987). Hence, it is necessary to match experimental groups tightly as done here if it is theoretically important.

### 1.3 Further tests

*WM*: Children were administered five subtests of the Automated Working Memory Assessment (AWMA; Alloway, 2007); which included two measures of verbal STM: Digit Span and Word Recall; one measure of visuo-spatial STM: Dot Matrix; one measure of verbal WM: Listening Span; and one measure of visuo-spatial WM: Odd One Out (OOO). Raw and standardized *recall* scores for all subtests, as well as *processing* scores for Listening Span and OOO were measured.

*Trail-making task*: Trail-making tests A and B were administered. Each received a score (2 = no errors or self corrected, 1 = one error, 0 = two or more errors) and solution speed was measured in seconds.

*Mental rotation*: Three separate worksheets with different stimuli types (objects/animals, letters and hands) were presented to the children; each worksheet had seven items. For each item within a worksheet, a target stimulus was presented, along with three comparison stimuli, two of which were mirror images and one was identical to the target. All three comparison images were rotated by various angles. The children were required to identify and circle the stimulus identical to the target. Children's accuracy and time to complete all seven items were recorded for each worksheet.

*Spatial symmetry*: Children were presented with two pages which contained six half drawn shapes against a grid background. A dashed line indicated the line of symmetry. Children were required to draw the other half of the shape for each item. Shapes (and lines of symmetry) were presented vertically on one page and horizontally on the other. The total time to complete the 12 shapes was recorded and the accuracy of items was scored with one point for every correct line segment.

### 1.4 Computerized experimental tasks

The following tasks were presented by the Presentation program of Neuro-behavioral Systems using a laptop computer. Unless described otherwise, RT and accuracy were recorded for all trials. See Supplementary methods for further details.

*Simple RT*: Children pressed a key in response to a white square which appeared after 1000, 2500 or 4000 msec (delay factor). There were 60 trials.

*Sustained attention*: Children were required to attend to a stimuli stream (letters) and to detect a target sequence (A B C) and to withhold responses to other sequences containing the target letters (‘deceiver trials’; e.g., A B D) or sequences containing no target letters (‘non-target trials’; e.g., D H F). The number of hits and misses for targets, the RT for target hits, the number of correct rejections and false alarms for deceivers and non-target trials, were recorded. Children were presented with 80 triads of the three different trial types.

*Stop-signal task*: A white arrow, pointing left or right, was shown on a black background in the middle of the screen. The arrow was either followed by a sound, the stop signal, or there was no sound. Children were required to indicate the direction of the arrow using a key press during ‘go’ trials, and to withhold their responses during ‘stop’ trials. The ratio of ‘go’ and ‘stop’ trials was 2:1. For each trial we measured RT, Stop signal RT (defined as the RT – average stop signal delay), and the number of times the child responded to the arrow incorrectly. 180 trials were presented.

*Animal Stroop*: Stimuli were colored pictures of two animals. Children were instructed to press a button on the keyboard on the side corresponding to the animal which was bigger in real life (Szűcs et al., 2009; Bryce et al., 2011). In the congruent condition the animal which was larger in real life was presented in a larger picture than the animal which was smaller in real life. In the incongruent condition the animal which was larger in real life was presented in a smaller picture than the animal which was smaller in real life. 96 trials were presented.

*Numerical magnitude comparison Stroop task*: Stimuli were pairs of white Arabic digits shown simultaneously on black background. There were four possible number pairs, with two different numerical distances. Children were instructed to decide which item of the pair was numerically larger than the other one and pressed a key where they detected the numerically larger stimulus. Numerical and physical size information could be neutral, congruent or incongruent with each other in equal proportions (congruency factor). In the congruent condition the numerically larger digit was also physically larger than the other one. In the incongruent condition the numerically larger digit was physically smaller than the other one. In the neutral condition both digits were of the same physical size. Numerical distance between stimuli was either 1 or 7 (numerical distance factor). 192 trials were presented.

*Physical size comparison Stroop task*: This task was identical to the numerical magnitude Stroop task, with the exception that the task was to respond to the physically larger stimulus. In neutral trials the digits differed in physical size but were numerically identical. 192 trials were presented.

*Subitizing*: Arrays containing one to six black dots appeared on a white background and children were instructed to say the number of dots as quickly as possible. Dot stimuli were presented in canonical and, where possible, non-canonical arrangements. RTs were measured using a voice-key. 60 trials were presented.

*Symbolic magnitude comparison*: Children decided whether visually presented digits were smaller or larger than 5. Children pressed a button on the keyboard with their left hand if the number was smaller than 5 and another button with their right hand if the number was larger than 5. 80 trials were presented.

*Non-symbolic magnitude comparison*: Two sets of black dots were presented simultaneously on a white background. The children's task was to decide which set contained more dots and press the button on the side of the larger set. Dot size was varied between sets. The following factors were manipulated in the construction of the stimuli sets: (1) The ratio of the number of dots in the two sets (1:2, 3:5, 2:3); (2) The numerical distance between the number of dots in the two sets; (3) The type of the physical control variable; (4) The congruity of physical control variables and numerosity; (5) The overall numerical sum of items in a display. See Supplementary methods for further details. 128 trials were presented.

### 1.5 Statistics

First, DD minus control difference scores were computed for tests and for the most important experimental contrasts (see details in Supplementary material): simple RT; animal Stroop task congruency; numerical and physical size Stroop task numerical distance effect, facilitation and interference; subitizing slope (numbers 1–3), counting slope (numbers 4–6); non-symbolic comparison slope and congruency effect, symbolic comparison slope; Stop-signal task hit and correct rejection performance.

Difference score data was assessed by robust non-parametric permutation testing (Ludbrook and Dudley, 1998). Dependent variables were test scores, accuracy and median RT. Procedure followed Chihara and Hesterberg (2011). DD minus control group difference scores were computed for all measures and the whole pool of participants were randomly divided into two groups of 12 participants one million times. Two-tailed significance values were determined with six decimal digits precision. In order to provide an estimate of effect size, empirical 95% confidence intervals for difference scores were also determined by bootstrap resampling producing one million bootstrap samples with replacement for each group.

Second, all experimental data was also analyzed by analyses of variance (ANOVAs) with full factorial designs. Third, while permutation tests provide extremely stringent criteria and groups were perfectly matched on several factors, difference scores showing significant permutation testing effects were nevertheless further analyzed by ANCOVAs with a group factor and with covariates of verbal intelligence (WISC Vocabulary), non-verbal intelligence (Raven) and simple RT speed (median RT from the Simple RT task). With matched groups this procedure can further increase power (Miller and Chapman, 2001). Fourth, simultaneous multiple regression analysis was used to study the relative weight of variables which significantly discriminated between the DD and control groups and were correlated with maths performance (the mean of the MaLT and WIAT Numerical Operations scales). Regressions are described further in Results. Analyses were programmed in Matlab.

## 2 Results

### 2.1 Memory

**Fig. 2:**
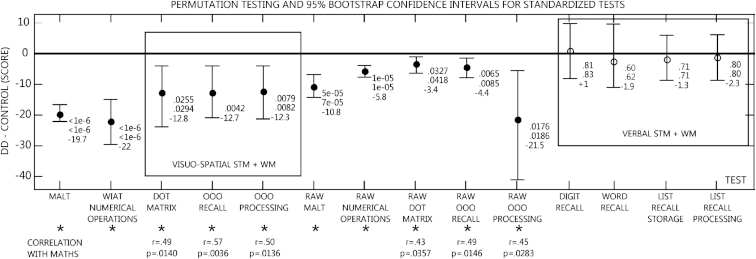
Permutation test results and bootstrap confidence intervals for standardized test scores. DD minus control difference scores are shown. Circles show the mean DD minus control group differences. Filled circles and stars denote significant group differences. Bars represent bootstrapped 95% confidence intervals. The upper number next to circles represents the permutation test *p* value for group differences. The middle number represents the independent sample *t*-test *p* value. The bottom number is the mean effect size in test score. Both standard and raw scores are shown for tests with significant effects. Only standard scores are shown for tests with non-significant effects (verbal STM + WM). Significant correlations between test scores and maths performance are shown below stars.

Fig. 2 summarizes significant DD versus control group differences in standardized test scores. The two groups differed on measures of visuo-spatial STM (Dot Matrix) and WM (OOO Recall, OOO Processing). 95% bootstrapped confidence intervals were robustly below zero for each measure showing a significant group difference (i.e., the DD group performed worse than the control group). For comparison, means and confidence intervals for non-significant verbal STM (Digit Recall, Word Recall) and WM measures (Listening Recall and Processing) are also presented. Table 1 shows *F* and *p* values from ANCOVAs for significant tests taking verbal IQ, non-verbal IQ and processing speed as covariates.

### 2.2 Accuracy measures

**Fig. 3:**
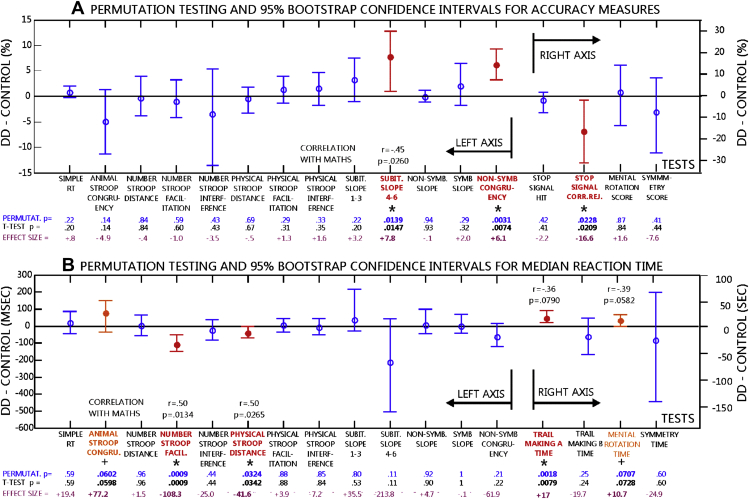
Permutation test results and bootstrap confidence intervals for (A) accuracy and (B) median RT measures. DD minus control difference scores are shown. Permutation and *t*-test *p* values and mean effect sizes (accuracy and RT) are shown below figures. Significant correlations between measures and maths performance are shown in the figure if significant or marginal (*r* and *p* values). Significant group differences are marked by red bars, text and stars. Marginal results are marked by orange bars, text and crosses.

Fig. 3A summarizes main DD minus control group differences in accuracy. The figure shows permutation and *t*-test statistics outcomes and bootstrapped 95% confidence intervals for effect sizes. Detailed experimental results and results of factorial ANOVAs are shown in Supplementary Fig. 1. Table 2 shows *F* and *p* values from ANCOVAs for significant tests taking verbal IQ, non-verbal IQ and processing speed as covariates. There were significant group differences in three measures. First, in the subitizing task counting-range slope was less steep in DD than in controls in the 4–6 number range. This was due to a larger drop in accuracy for number 6 in controls than in DD (see star in Supplementary Fig. 1D). Second, there was a larger congruency effect in DD than in control participants in non-symbolic magnitude comparison (see star in Supplementary Fig. 1F). Third, correct rejection performance was worse in DD than in controls in the Stop-signal task (see star in Supplementary Fig. 1E). In ANOVAS there was an additional marginal group × congruency interaction in the animal size Stroop task due to a marginally larger congruency effect in DD than in controls (Supplementary Fig. 1B). The trail-making task was scored on a 0–2 scale. Accuracy was practically the same in both groups in both trail-making A/B: All DD participants and all but one control scored maximum on trail-making A (a single control scored 0). Scores were also matched on trail-making B (number of DD/Control participants with particular scores: Score 2: 8/7; Score 1: 2/2; Score 0: 2/3). Importantly, both permutation testing and confidence interval estimation showed that symbolic and non-symbolic slope was a highly non-discriminative parameter between groups. Fig. 3 shows effect sizes. In detail, in the non-symbolic discrimination task the mean ratio effect was −1.75 ± .5% (mean and SE; accuracy for each ratio: 97.2 ± 1.1, 95.6 ± 1.4 and 93.7 ± 1.6%) in the DD group and −1.70 ± .4% in the control group (accuracy for each ratio: 97.7 ± .9, 95.2 ± 1.8 and 94.3 ± 1.8%). In the symbolic discrimination task the mean distance effect was −3.26 ± 1.4% (distance 1 minus distance 4) in the DD group and −5.24 ± 1.4% in the control group (accuracy for each level of distance: DD: 91.5 ± 1.9 and 94.8% ± 1.3; controls: 89.0 ± 2.3 and 94.2 ± 1.6%).

### 2.3 Median RT

Fig. 3B summarizes main findings in RT with permutation testing and *t* statistics and bootstrapped 95% confidence intervals for effect sizes. Detailed experimental results and results of factorial ANOVAs are shown in Supplementary Fig. 2. Table 3 shows *F* and *p* values from ANCOVAs for significant tests taking verbal IQ, non-verbal IQ and processing speed as covariates. There were significant group differences in four measures. First, there was a larger facilitation effect in the numerical Stroop task in DD than in control participants (Supplementary Fig. 2G). The negative effect means that RT sped up more in the congruent relative to the neutral condition in DD than in control participants. This means that task-irrelevant physical size information had a larger effect on RT in DD than in controls. As optimal task performance requires focusing on the task-relevant numerical dimension, larger facilitation from physical size information reflects the intrusion of the task-irrelevant stimulus dimension into processing. Hence, this effect is a marker of failure to inhibit the task-irrelevant stimulus dimension. Second, there was a larger distance effect in DD than in controls in the physical size decision Stroop task (Supplementary Fig. 2H). This means that task-irrelevant numerical information had a larger effect on RT in DD than in controls. Third and fourth, trail-making A (Mean/SE: DD = 58.3 ± 5.4 sec; Control = 41.3 ± 2.0 sec) and mental rotation (DD = 66.7 ± 4.4 sec; Control = 56.0 ± 3.5 sec) solution times were longer in DD than in controls. Further, there was a marginally larger congruency effect in the animal size decision Stroop task in DD than in controls (Supplementary Fig. 2B). This means that task-irrelevant physical size information had marginally larger effect on RT in DD than in controls. Again, both permutation testing and confidence interval estimation showed that symbolic and non-symbolic slope was a highly non-discriminative parameter between groups. There were no effects in coefficient of variation (see Supplementary Fig. 3).

### 2.4 Regression

Regression analysis was used to study the relative weight of variables which significantly discriminated between DD and control and correlated with maths performance. The three visuo-spatial memory measures (Dot Matrix, OOO Recall and Processing) were averaged to form a single ‘Visuo-spatial memory’ measure. The RT facilitation effect from the numerical Stroop task and the RT distance effect from the physical size decision Stroop task were averaged to form an ‘Inhibition’ score because only these measures showed a significant correlation with maths performance (see correlations in Figs. 2 and 3). The counting-range slope from accuracy data was also used because this also showed a significant correlation with maths performance. Correlations between the above variables and maths scores are shown in Table 4. The above three variables were entered into the analysis simultaneously. The regression had a significant fit [*R*^2^ = .583, *F*(20,3) = 9.30, *p* < .0001]. Visuo-spatial WM [Standardized Beta (*β*) = .48, *t*(20) = 3.2, *p* = .0045] was a significant predictor and Inhibition [*β* = .36, *t*(20) = 2.06, *p* = .0522] was a marginally significant predictor. Subitizing slope was a non-significant predictor [*β* = −.17, *t*(20) = −1.02, *p* = .31]. When only Visuo-spatial WM and Inhibition were entered into the regression the overall fit remained unchanged: [*R*^2^ = .561, *F*(21,2) = 13.39, *p* < .0001]. Visuo-spatial WM: *β* = .48, *t*(21) = 3.24, *p* = .0039. Inhibition: *β* = .45, *t*(21) = 3.00, *p* = .0068. When verbal IQ (WISC Vocabulary), Raven score and processing speed were added to the regression, the overall fit increased [*R*^2^ = .633, *F*(20,3) = 9.30, *p* < .0001] but only Visuo-spatial WM [*β* = .61, *t*(20) = 3.60, *p* = .0020] and Inhibition [*β* = .35, *t*(20) = 2.18, *p* = .0421] were individually significant predictors. Subitizing slope remained a non-significant predictor when it was entered into the regression with only the Inhibition ability measure [*R*^2^ = .368, *F*(21,2) = 6.13, *p* = .0080; Subitizing: *β* = −.19, *p* = .34; Inhibition: *β* = .48, *p* = .0297].

## 3 Discussion

We have contrasted five theories of DD using several measures of the MR theory and alternatives. We found robust evidence for impaired *visuo-spatial WM and STM* in DD and also found evidence for impaired *inhibition* function in DD. Data did not support the MR theory of DD.

### 3.1 There were robust visuo-spatial WM and visuo-spatial STM impairments in DD

In contrast, verbal STM/WM were intact including both digit and word span. Several studies reported similar dissociation between spatial and verbal STM/WM in DD (McLean and Hitch, 1999; Andersson and Ostergren, 2012; Schuchardt et al., 2008; Ashkenazi et al., 2012; Passolunghi and Mammarella, 2010). Other studies reported impaired verbal STM/WM in DD (e.g., Geary et al., 1991, 2012). A potential dissociating feature seems to be that studies not reporting verbal WM differences noted that they attempted to match DD and control groups on reading and/or verbal performance (McLean and Hitch, 1999; van der Sluis et al., 2005; Schuchardt et al., 2008; Andersson and Ostergren, 2012; Ashkenazi et al., 2012; Passolunghi and Mammarella, 2010). Our DD group also only included children with pure DD with no dyslexia and with normal reading/verbal IQ. This probably explains the lack of verbal memory differences. In fact, Schuchardt et al. (2008) tested both visual and spatial STM in DD, dyslexic, DD + dyslexic and normal populations and found only visual STM impairment in DD and only verbal STM impairment in dyslexics. Hence, it seems that when reading and verbal function is preserved, that is, in pure DD, a crucial impairment concerns visuo-spatial WM and/or STM.

At least three neuro-imaging studies provide supporting evidence to our findings. Rotzer et al. (2009) demonstrated weaker IPS activation in a spatial WM task in DD than in controls. Rykhlevskaia et al. (2009) reported reduced gray matter density in DD not only in the IPS but also in the fusiform, lingual, parahippocampal gyri and in the hippocampus, areas which may be related to encoding complex visual stimuli. Davis et al. (2009) did not find any IPS differences between DD and controls in an approximate calculation task but reported differences in various brain regions associated with WM and cognitive control functions. Visuo-spatial memory probably provides a mental workspace for various transformations and operations crucial for mathematics. Visuo-spatial strategies and heuristics can be used even in seemingly non-visual tasks, e.g., when adding or subtracting numbers, operations and operands can be imagined/conceptualized along a number line. Our and other findings reviewed above suggest that this important general visuo-spatial workspace does not function properly in DD.

An important question concerns that most studies reported only visual STM (McLean and Hitch, 1999; van der Sluis et al., 2005; Schuchardt et al., 2008; Ashkenazi et al., 2012; Passolunghi and Mammarella, 2010) impairment in DD while only one of the above studies reported WM impairment (Andersson and Ostergren, 2012). A conspicuous factor explaining this discrepancy is that in fact only Andersson and Ostergren (2012) used WM tasks in the visual modality. The other studies did not measure specific visuo-spatial WM because they relied on the classical WM model of Baddeley (1986) which assumes that the so-called central executive function underlying WM performance is amodal. Hence, most studies measured WM (central executive) performance with purely verbal tasks or some tasks may have included spatial elements but with a strong simultaneous verbal component (Schuchardt et al., 2008). However, there is accumulating evidence that WM function may in fact dissociate by stimulus modality and cannot be considered dependent on amodal central executive resources (Shah and Miyake, 1996; Jarvis and Gathercole, 2003). In fact, our study provides further evidence for dissociation between verbal and visual WM systems. Hence, it seems crucial to measure STM and WM capacity separately in the verbal and visual modalities.

### 3.2 Five findings point to impaired inhibitory function in DD

There were larger congruency effects in DD than in controls in the non-symbolic magnitude decision task (from the intrusion of non-numerical parameters) and in the animal Stroop task (from the intrusion of physical size). In the numerical Stroop task DD were more affected by task-irrelevant physical size. In the physical size decision Stroop task DD were more affected by task-irrelevant numerical magnitude and hence had a larger automatic numerical distance effect than controls. First, this finding demonstrates that the automatic processing of numerical magnitude happened in DD. Second, it is unlikely that DD had a larger involuntary distance effect than controls because DD processed magnitude more efficiently than controls. Rather, in the context of generally larger congruency effects in DD findings suggest that DD could not resist the intrusion of task-irrelevant stimulus dimensions as efficiently as controls. Similar data was reported by Landerl and Kolle (2009) who found larger unit/decade compatibility effects in DD than in controls and concluded that this was due to worse interference suppression in DD than in controls (again, the unlikely alternative explanation could be that DD are better in interpreting multi-digit numbers than controls). They also reported a smaller size congruity effect in DD than in controls in the physical size decision Stroop task. Here we did not find such an effect while using more than five times as many trials (192 *vs* 36) than Landerl and Kolle (2009). The difference may also be due to the fact that the DD group in Landerl and Kolle's (2009) study performed worse than controls in word and non-word reading and the Block Design tasks. The poorer correct rejection performance in the Stop-signal task suggests difficulty in withholding an inaccurate response.

Overall, our data from five different experiments suggests that DD were more susceptible to the effect of task-irrelevant information than controls. Similar to our findings, interference suppression weakness was reported in DD children/adults and in children with weak mathematical skills in the Wisconsin Card Sorting Task (Bull et al., 1999) and arithmetic tasks (Pasolunghi et al., 1999; Passolunghi and Siegel, 2004; De Visscher and Noël, 2013). In addition, tasks with interference suppression demands have been shown to be strongly related to mathematical development (e.g., Bull and Scerif, 2011; Espy et al., 2004; Blair and Razza, 2007; Swanson, 2011; Marzocchi et al., 2002). Inhibition function impairment could lead to mathematical problems because Numerical Operations require the temporal and spatial (in imagination) coordination of several processes and the retrieval of several highly similar facts – impaired inhibition probably interferes with the organization of these processes. In addition, various theories of WM function assume that inhibitory processes and specifically interference suppression play an important role, and/or are crucial components of the central executive function of WM (e.g., Hasher and Zacks, 1988; May et al., 1999; Miyake et al., 2000; Caretti et al., 2004). Hence, we suggest that the WM and inhibition impairments detected in our study may be related to each other and the inhibition impairment may have led to impaired visuo-spatial WM performance. Were this hypothesis true, DD could be attributed to the specific impairment of visuo-spatial STM and to the specific impairment of the inhibitory processes crucial to visuo-spatial central executive WM function. In fact, the IPS has been demonstrated to be involved in interference resolution (Mecklinger et al., 2003; Cieslik et al., 2011). Hence, DD versus control differences in at least some functional and structural MRI IPS data may be related to differences in interference resolution rather than to MR/ANS function.

Our results seem to fit into a wider framework of data reported with regard to learning disabilities. Several studies found that children with poor reading comprehension show deficits in interference suppression in verbal WM tasks (De Beni et al., 1998; Pimperton and Nation, 2010) but not in visuo-spatial WM tasks (Pimperton and Nation, 2010). Interference suppression deficits in verbal WM tasks were also reported in children with ADHD (Cornoldi et al., 2001; Palladino, 2006; Palladino and Ferrari, 2013). Importantly, while all the above studies found decreased verbal WM performance in children with dyslexia and ADHD, our study did not find any general verbal WM difference between DD and control children. In contrast, here we found a robust visuo-spatial WM difference. On the other hand, Pasolunghi et al. (1999) and Passolunghi and Siegel (2004) did report both verbal WM differences and interference suppression difficulties in DD children. Both of these studies matched DD and control children in verbal IQ and Passolunghi and Siegel (2004) also matched reading performance, and the studies used DD diagnosis cutoff scores at the 20th and 30th percentiles, respectively. Hence, diagnosis was more permissive than in our study and a further difference seems to be that diagnosis relied on a standardized test in which eight out of 12 problems were word problems (e.g., ‘On Pascoli Street there are 45 shops. 3/5 of them sell clothes. How many clothes shops are there in Pascoli Street?’; Pasolunghi et al., 1999; p. 781). In contrast, our study relied on two tests with overwhelmingly Arabic digit computational problems. Hence, speculatively, perhaps the content of the tests used to identify the DD children affected results. In fact, Passolunghi and Siegel (2004) report a .38SD reading score difference between their DD and control populations. Assuming standard deviation (SD) = 15 this is equivalent to 5.7 score difference between groups. As shown in Fig. 1 in our sample differences in reading scores ranged between .2 and 2 scores, so DD and control populations were slightly better matched which may affect verbal WM results. Further, Pasolunghi et al. (1999) and Passolunghi and Siegel (2004) did not measure visual STM and WM function. Overall, this comparison points to the importance of matching diagnostic instruments across studies and testing both verbal and visual WM. In addition, future studies should explore the exact nature of potential interference suppression deficits in DD in visuo-spatial STM/WM tasks and investigate whether interference suppression deficits in different learning disabilities are the consequence of similar impaired mechanisms manifesting in different modalities.

### 3.3 Preserved but slow spatial processing and slow trail-making speed in DD may be secondary to WM/inhibition impairment

Accuracy equaled in DD and controls in the spatial symmetry task and in the mental rotation task. We detected slower solution times in DD than in controls on the trail-making A task, which confirms some previous findings (McLean and Hitch, 1999; Soltész et al., 2007; Andersson, 2010), as well as on the mental rotation task. The accurate performance on the symmetry and rotation tasks suggests that spatial skills were available to DD albeit at a slower speed than to controls. Hence, we conclude that slower rotation speed and the slow trail-making performance (this task is usually thought to be very dependent on WM central executive function) relate to WM and inhibition function impairment in DD.

### 3.4 None of our findings support the MR theory

The lack of positive findings with regard to the MR theory of DD is in sharp contrast with robust visuo-spatial STM/WM and inhibition-related findings. We have a number of reasons to assume that the lack of group × measure interactions in MR measures was not due to lack of power. First, our study clearly had enough power to detect *all* expected experimental effects in *all nine* experiments. Most importantly, we detected *all* expected ratio and congruency effects in the symbolic and non-symbolic magnitude discrimination tasks and *detected other* group × measure interactions at good significance levels.

Second, in order to achieve high intra-individual power our study deliberately had a large number of trials in each experiment. There were 40 trials for each level of symbolic numerical distance in the symbolic discrimination task (80 stimuli all together) and 40 trials for each level of ratio in the non-symbolic discrimination task (120 stimuli all together). That is, across the study we collected 12 × 40 = 480 trials for each ratio level in the DD group. In comparison to studies with positive MR results our study had 1.66–4 times as many trials per ratio level than other studies: Price et al. (2007) presented 12 trials per ratio level (24 stimuli, eight DD children, i.e., 96 trials for each ratio across the whole study), Mazzocco et al. (2011) used 20 trials per ratio level (80 stimuli, 10 DD children, i.e., 200 trials per ratio level across the whole study), Mussolin et al. (2010a, 2010b) used 24 trials per ratio level (96 stimuli for each presentation format, 15 DD children, 360 trials per ratio level for each presentation format across the whole study), Piazza et al. (2010) used 10 trials per ratio level (80 stimuli, 23 DD children including 12 dyslexic children, i.e., 230 trials per ratio level across the study). In addition our study had 12 DD children which is more than the number of DD children in two out of the above four studies. Even when factoring in the larger number of DD children in the two remaining studies (Mussolin et al., 2010a, 2010b; Piazza et al., 2010) our study collected 1.33–2.08 times more trials per ratio level for each presentation format than other studies. This is advantageous because the larger number of trials effectively suppresses the amount of noise inherent to the data which increases power.

Third, the impaired MR theory predicts that ratio effects in non-symbolic number discrimination will differ in DD relative to controls (Piazza et al., 2010; Mazzocco et al., 2011; Price et al., 2007). In our study the between group difference in the mean ratio effect was .1%. In a similar non-symbolic number discrimination task Price et al. (2007) observed a 2.5% difference between groups in the ratio effect with the DD group showing a larger effect than controls because DD children were less accurate than controls at close ratios (close *vs* far ratio difference in controls: 3.87%, DD: 6.37%; accuracy for close *vs* far ratios in controls: 95.75% vs. 99.62%. In DD: 92.75% vs. 99.12%). In that study the standard deviation of the error data was about 1.65% and the group difference in the ratio effect was about 1.51SD. For the 12 subjects in our study this gives a Power estimate of Power > .99. In our study comparable accuracy values were found (both controls and DD: 93.7–97.7%) with a ratio effect of comparable effect size (1.7%) with larger SD (2.97%). However, considering the similar size of the overall accuracy and distance effects in relation to Price et al. (2007), in our study the .1% between group ratio effect difference we found can be considered practically zero. This is confirmed by the fact that the bootstrap 95% confidence interval of the non-symbolic comparison ratio effect was clearly focused on zero (see Fig. 3.), the very small confidence intervals were approximately symmetric around zero and SEs were very small, about .4%. All the above suggests that there was not much variability or directional bias in our data and that there was not even an indication of a difference in the ratio effect between the groups.

Fourth, regarding the symbolic magnitude comparison task the mean of the between group difference was 2% and the SD of the data was about 5.71%. The DD group showed a smaller absolute value distance effect than the control group (3.26% *vs* 5.24%). Crucially, DD actually showed slightly better performance on the task than the controls while RTs were practically identical. This makes it unlikely that DD had impaired access to MRs in this task. Nevertheless, in the data from the Arabic number comparison task of Mussolin et al. (2010a, 2010b) the overall mean distance effect (calculated for all four ratios used; see ibid. Table 2) was actually exactly the same in the control and DD groups (2.76%) and the difference between the most extreme distance levels was also the same in both groups (8.3%). The DD and the control group showed a difference because the closest levels of distance differed more in the DD than in the control group. However, this means that the DD group was .6% less accurate at the closest level of distance while it was actually 1.1% more accurate than the controls at the second closest level of distance. The difference between the groups was 1.7% (controls: 2.7%; DD: 4.4%) and the SD of the data was about 1.75% (this is not very clear as the table reports exactly the same standard deviation values for both groups which is probably a mistake). Hence, the group difference was .97SD. For our 12 subjects such an effect size would give Power > .99. (It is to note that crucial analysis results in Mussolin et al. (2010) relied on trials collected from 5 different stimulus formats (5 × 24 = 120 trials for each level of distance) rather than from an individual stimulus format.) However, we only measured a 2% (.33SD) between group difference in the distance effect. In addition, as noted above, the somewhat higher accuracy in the DD than in the control group also makes it unlikely that our DD group had problems with accessing the magnitude of single Arabic digits.

Fifth, it is important to emphasize the difference between the robustness (large effect size) of WM and inhibition results in contrast to MR-related results. Our data definitely did not give any indication of a non-symbolic ratio effect discrepancy between groups and while it is naturally hard to exclude that *perhaps* a significant symbolic distance effect difference could have emerged by using more trials from more participants, WM and inhibition-related findings appeared clearly. In contrast, any *potential* MR-related effects seem harder to detect and fragile relative to the variability in data. The robustness of WM/inhibition results is an extremely important factor to consider when it comes to testing theories and diagnosing children at the individual level and remediation of DD.

Sixth, our study joins several studies with negative results with regard to the MR theory of DD. To date eight studies could not detect any distance/ratio effect discrepancy between DD and controls (Landerl et al., 2004; Kucian et al., 2006, 2011; Rousselle and Noël, 2007; Soltész et al., 2007; Landerl and Kolle, 2009; Mussolin et al., 2010b; Kovas et al., 2009) while four studies reported such a difference (Price et al., 2007; Mussolin et al., 2010a; Piazza et al., 2010; Mazzocco et al., 2011). However, as noted before, none of these four studies used non-numerical control tasks and their crucial non-symbolic number comparison diagnostic task is inevitably confounded by visual stimulus parameters (Gebuis and Reynvoet, 2011, 2012) which particularly seriously affects the computation of ‘w’, a proposed measure of the MR (Szűcs et al. 2013). It is also important to note that sometimes simply worse accuracy on MR tasks in DD than controls is considered evidence for impaired MR in DD. However, obviously, worse accuracy (especially when there is no control task) can appear for various reasons (see e.g. Szűcs et al., 2013). Hence, decreased accuracy cannot be considered evidence for specific MR impairment. Overall, we conclude that DD and control groups were practically indistinguishable on measures of the MR while other tasks strongly and clearly discriminated these groups.

### 3.5 Subitizing and counting

The only piece of data from our study which could perhaps call for number-specific explanations is that the counting-range slope (4–6 number range) in accuracy in the subitizing task was less steep in DD than in controls. However, first, this finding appeared because DD children were more accurate for number 6 than controls. Second, there were no effects in RT which is usually considered the main measure in subitizing tasks. Third, when counting-range slope accuracy and the Inhibition measure were entered into a regression together, counting-range slope was a non-significant predictor of mathematical performance. When only WM and Inhibition were entered into regression, the model fit remained practically unchanged. WM and Inhibition were significant predictors even when entered with verbal and non-verbal IQ measures and with processing speed. WM and Inhibition scores were not correlated which suggests their independence. In contrast, counting-range slope correlated with Inhibition and remained a non-significant predictor when inhibition was included in the regression. Hence, as no other MR-related measure discriminated between groups, counting-range slope findings seem to be related to inhibition ability and not to MR function.

### 3.6 Diagnosis issues

It is important to point out that there is substantial variation across studies in defining children with DD due to the fact that there is no agreed definition of DD. The range of cutoffs used to define DD in demographic studies ranges from performance below the 3rd percentile to performance below the 25th percentile (2SD–.68SD below the mean; for review see Devine et al., 2013). Here we used very stringent criteria to assure that children only had mathematical difficulties. We screened 1004 children and diagnosed DD if performance on two standardized mathematical measures was worse than 1SD while there was no ADHD and dyslexia, verbal IQ/reading was normal on four different tests and non-verbal IQ was normal on two tests. For example, Price et al. (2007) screened 55 children and WISC block-design performance differed by more than 1SD between DD and controls. In Piazza et al. (2010) about half the DD group was diagnosed with dyslexia. Mussolin et al. (2010a) screened 187 children and diagnosed DD if performance was worse than −1SD (15th percentile) on a multiplication test. However, multiplication relies heavily on verbal memory (Ashcraft, 1982). Mazzocco et al. (2011) screened 161 children and diagnosed 10 children below −1.3SD (10th percentile) with DD and children below −.65SD (25th percentile) as low maths achievers without using any other criteria. Various tests were used as covariates in analyses. However, the tests were recorded in various years during a 7-year long period and as noted above, ANCOVAs cannot ‘correct for’ major differences along independent variables (Miller and Chapman, 2001; Porter and Raudenbush, 1987). Obviously, definition and measurement discrepancies can contribute to disagreeing findings across studies.

### 3.7 Conclusion

In summary, there is evidence that IPS morphology and perhaps function differ between DD and control participants (Isaacs et al., 2001; Rotzer et al., 2008; Price et al., 2007; Mussolin et al., 2010b). However, there is insufficient evidence for the argument that IPS dysfunction in DD can be linked to MR dysfunction: (1) Only one out of six fMRI studies found supporting behavioral data (Price et al., 2007). (2) The frequently used dot comparison task is seriously compromised by non-numerical confounds (Gebuis and Reynvoet, 2011; 2012; Szűcs et al., 2013). (3) Several behavioral and fMRI DD studies focusing on the MR theory of DD do not have non-numerical control conditions. (4) Adding to several negative findings (see above) our study used several measures of the MR but could not detect any clear MR impairment effects in DD. The fallibility of evidence for the MR theory of DD is in sharp contrast with the robust nature of the visuo-spatial STM/WM difference between DD and control groups in our data which is in agreement with various studies. Verbal WM/STM is probably only impaired if DD is accompanied by reading/verbal difficulties (e.g., with dyslexia).

We conclude that the MR theory of DD which is currently dominant in neuroscience research is insufficient to explain pure DD. Hence, there is a need for a paradigm shift in DD research; neuro-imaging studies should now take alternative theories of DD, defined by extensive behavioral research, seriously. Crucially, rather than aiming at reconfirming a single theory of DD, studies should test theories against each other. Our data suggests that the most robust dysfunction in DD is that of visuo-spatial STM and WM with the impairment of inhibitory function (interference suppression). Both of these functions have been linked to the IPS. Hence, we suggest that IPS dysfunction in DD is probably related to WM and inhibition impairment. We hypothesize that the WM and inhibition impairments are related to each other and the inhibition function impairment reflects the disruption of a crucial processes of central executive memory function. That is, pure DD could be characterized by the specific impairment of visuo-spatial STM and by the specific impairment of the inhibitory processes crucial to visuo-spatial central executive memory function resulting in poor WM. Future imaging studies of DD should take these cognitive functions into account. Intervention studies could explore whether the above functions can be improved in DD. Spatial processing seems intact in DD albeit slowly accessible which is probably a consequence of memory/inhibition impairment.

## Figures and Tables

**Figure:**
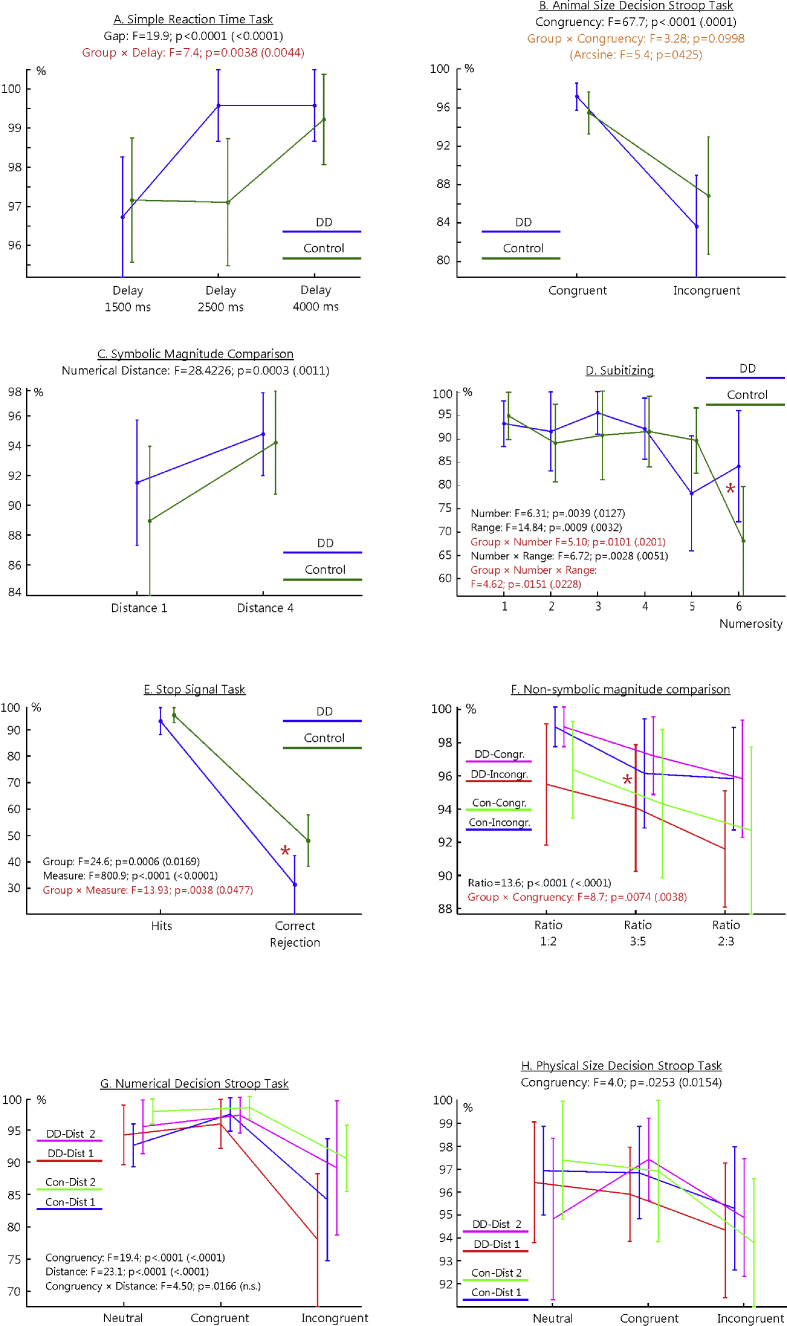
Supplementary Fig. 1. Factorial ANOVA results for accuracy data. *p* values in parentheses report additional analyses on arcsine transformed data. Significant results are embedded in figures. For interpretation refer to text.

**Figure:**
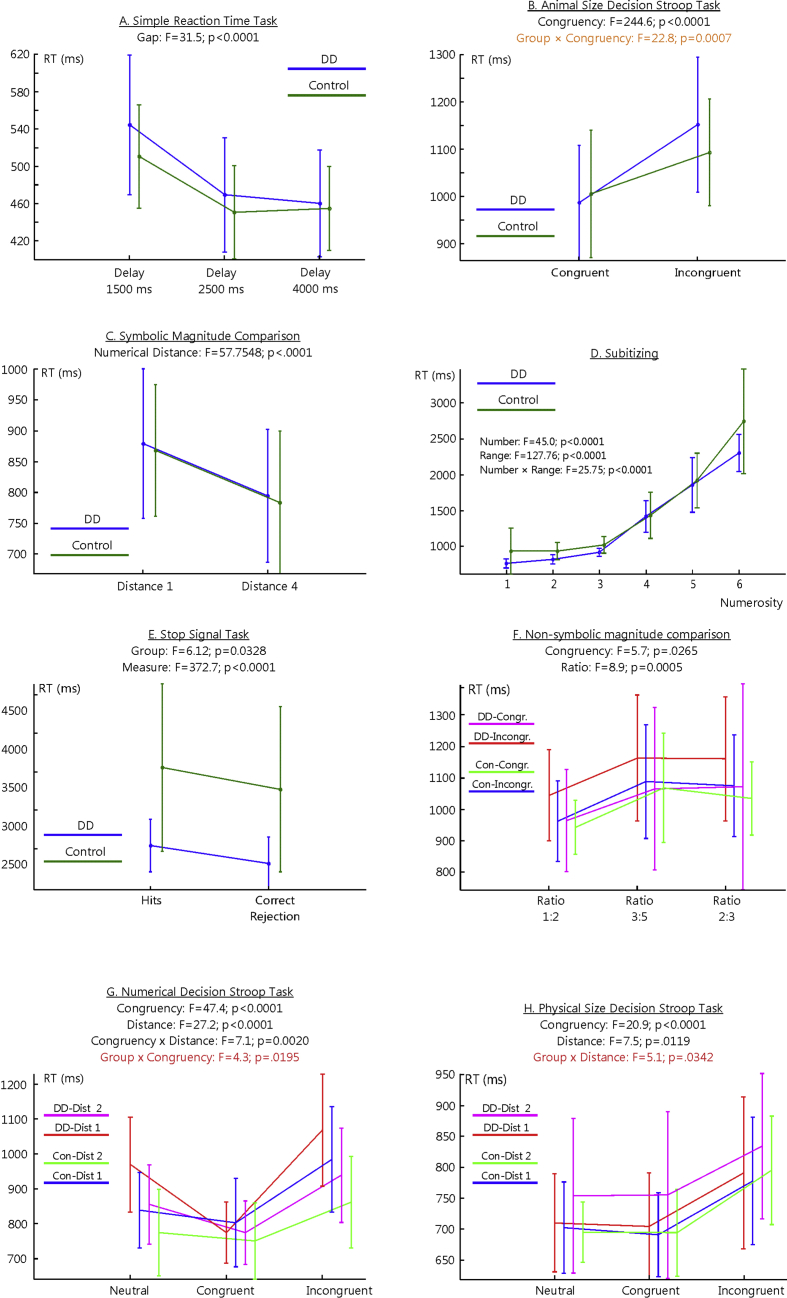
Supplementary Fig. 2. Factorial ANOVA results for median reaction time data. Significant results are embedded in figures. For interpretation refer to text.

**Figure:**
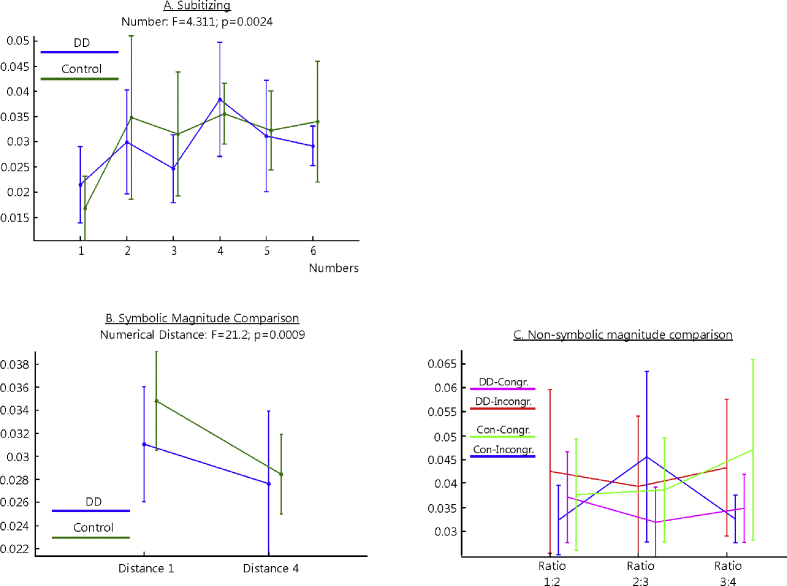
Supplementary Fig. 3. Factorial ANOVA results for the coefficient of variation measure from median reaction time data.

**Table 1:** ANCOVA results for WM tests.

	Dot Matrix	OOO recall	OOO processing	Raw dot matrix	Raw OOO recall	Raw OOO processing
Correcting for verbal IQ − *F*(1,21)=	5.13	9.89	7.88	4.23	8.19	6.05
*p* value	**.0348**	**.0051**	**.0108**	***.0529***	**.0096**	**.0232**
Correcting for non-verbal IQ (Raven) − *F*(1,21)=	5.69	15.18	13.20	6.15	17.73	13.66
*p* value	**.027**	**.0009**	**.0016**	**.0221**	**.0004**	**.0014**
Correcting for processing speed (Simple RT task) − *F*(1,21)=	5.45	7.82	6.47	4.81	6.23	4.72
*p* value	**.03**	**.0111**	**.0193**	**.04**	**.0214**	**.0419**
Correcting for all three factors − *F*(1,19)=	7.21	14.41	12.18	8.1	15.14	10.58
*p* value	**.0146**	**.0012**	**.0024**	**.0103**	**.0009**	**.0041**

Significant *p* values are in bold. Marginally significant *p* values are in bold italics.

**Table 2:** ANCOVA results for accuracy measures.

	Subitizing slope 4–6	Non-symbolic comparison congruency effect	Stop-signal task correct rejection
Correcting for verbal IQ − *F*(1,21)=	7.86	9.33	7.62
*p* value	**.0109**	**.0062**	**.012**
Correcting for non-verbal IQ (Raven) − *F*(1,21)=	8.79	7.9	6.86
*p* value	**.0076**	**.0107**	**.0164**
Correcting for processing speed (Simple RT task) − *F*(1,21)=	7.01	8.45	6.53
*p* value	**.015**	**.0084**	**.0184**
Correcting for all three factors − *F*(1,19)=	9.49	7.88	5.69
*p* value	**.0061**	**.0112**	**.0276**

Significant *p* values are in bold. Marginally significant *p* values are in bold italics.

**Table 3:** ANCOVA results for RT measures.

	Animal Stroop	Number Stroop facilitation	Physical size Stroop distance effect	Trail-making A speed	Mental rotation speed
Correcting for verbal IQ − *F*(1,21)=	5.19	16.27	4.57	10.12	*3.71*
*p* value	**.0338**	**.0006**	**.0449**	**.0046**	***.0682***
Correcting for non-verbal IQ (Raven) − *F*(1,21)=	*4*	13.04	4.44	10.74	*3.53*
*p* value	***.0591***	**.0017**	**.0477**	**.0037**	***.0747***
Correcting for processing speed (Simple RT task) − *F*(1,21)=	4.39	12.96	4.94	8.02	*3.18*
*p* value	**.0489**	**.0018**	**.0378**	**.0102**	***.0895***
Correcting for all three factors − *F*(1,19)=	5.14	11.23	*3.74*	8.08	*3.75*
*p* value	**.035**	**.0033**	***.068***	**.0103**	***.0676***

Significant *p* values are in bold. Marginally significant *p* values are in bold italics.

**Table 4:** Correlation matrix for variables in the regression analysis. Marginal *p* values are in parentheses. The correlation of WISC Vocabulary (*p* = .31), Raven score (*p* = .77) and processing speed (*p* = .26) with maths was not significant.

		Maths	Counting-range slope	Visuo-spatial WM
Counting-range slope	*r*	−.45		
*p*	.0263		
Visuo-spatial WM	*r*	.61	*−.18*	
*p*	.0016	*n.s.* (*.4*)	
Inhibition	*r*	.58	−.53	*.27*
*p*	.0028	.0076	*n.s.* (*.2*)
