# Supplementary material for: Developmental dyscalculia is related to visuo-spatial memory and inhibition impairment
Source: Cortex. 2013 Nov;49(10):2674–88. doi: 10.1016/j.cortex.2013.06.007 (PMC3878850; doi:10.1016/j.cortex.2013.06.007)
Supplement: Supplementary file 1 [file mmc1.doc]

Supplementary Methods

Screening Phase 1

Sample

We tested 1,004 children (526 boys and 478 girls) ages 7 years 4 months to 10 years 1 month attending Year 3 (N=806 mean age = 8:1) and Year 4 (N= 198, mean age =9:1) of primary school. The participating schools were state primary schools located in Cambridgeshire (12 schools), Hertfordshire (8 schools) and Essex (2 schools), England. The schools comprised a mix of urban schools and outlying rural schools and the catchment populations of the schools were predominantly lower-middle class. The sample included children with statements of special educational needs and whose native language was not English. All children received parental consent to participate.

Tests

Mathematics test.

The mathematics tests used were the Mathematics Assessment for Learning and Teaching tests (MaLT) (Williams, 2005). The MaLT tests are group-administered written tests. The MaLT tests were developed in accordance with the National Curriculum and National Numeracy Strategy for England and Wales. Test items cover: counting and understanding number, knowing and using number facts, calculating, understanding shape, measurement, and handling data. The MaLT tests were standardized in 2005 with children from 120 schools throughout England and Wales (MaLT 8, α= 0.91; MaLT 9, α=0.93). Tests allowed 45 minutes for completion and scores were calculated out of a total of 45 points.

Reading test.

We used the Hodder Group Reading Test II (HGRT-II) (Vincent & Crumpler, 2007). The HGRT II level 1 was used for Year 3 pupils, and the HGRT II level 2 was used for Year 4 pupils. These multi-choice tests assess children’s reading of words, sentences and passages. The tests were standardized in 2005 with children from 111 schools throughout England and Wales (HGRT II level 1, α=0.96; HGRT II level 2, α=0.95). Each test has two parallel forms which were used in the present study to minimise copying. Tests allowed 30 minutes for completion. The raw score was out of 40 points for the level 1 test and 53 points for the level 2 test.

Procedure

Tests were administered to whole classes between March and December 2010. Classes typically completed both tests in one day, with a break in between the two tests. The order of test administration was counterbalanced across classes.

Children completed the tests under test-like conditions: the children’s tables were separated and children were discouraged from speaking or colluding with neighbouring children. At the beginning of the reading test the researchers explained the test instructions and administered two practice questions with the class before the test began. The children worked through the reading test without any input from the researchers or teachers except for explaining the test instructions again where required.

The mathematics assessments do not include practice questions, however the tests allow for invigilators to read the questions if required because the test items require reading and test performance should reflect mathematics ability rather than reading proficiency. Reading questions is also the convention for the administration of National Curriculum mathematics assessments in England and Wales. The test instructions were explained to the children before the test began and invigilators read the questions to the children where necessary.

**Screening Phase 2**

IQ

In order to estimate IQ we administered a two subtest short-form of the Wechsler Intelligence Scale for Children – 3^rd^ Edition (WISC-III, Wechsler, 1991) which included the Block Designs (non-verbal) and Vocabulary (verbal) subtests. This combination of subtests has the highest validity and reliability of the two-subtest forms of the WISC-III (r_tt_ = .91 r=.86; Table L-II, Sattler, 1992).

We also measured children’s non-verbal IQ using Raven’s Coloured Progressive Matrices (Raven’s – Educational: CPM, Raven, 2008).

Mathematics and Reading

Mathematics and reading achievement was measured using the Numerical Operations, Word Reading and Pseudoword Decoding subtests of the Wechsler Individual Achievement Test (WIAT-II UK, Wechsler, 2005). The Numerical Operations subtest includes assessment of counting, one to one correspondence, numerical identification and writing, calculation (addition, subtraction, multiplication, division) fractions, decimals and algebra. The Word Reading subtest assesses letter identification, phonological awareness, letter-sound awareness and the accuracy and automaticity of word recognition. Pseudoword decoding assesses phonological awareness and accuracy of word attack.

Further tests

Working memory

We assessed working memory using five subtests from the Automated Working Memory Assessment (AWMA, Alloway, 2007). The AWMA is a computer administered battery of tests which assess verbal short term memory (STM) and visuo-spatial STM and working memory (corresponding to the phonological loop, visuo-spatial sketch pad and central executive components of Baddeley and Hitch’s 1974 model of working memory, respectively).

Verbal working memory

*Short-term memory (STM):* Phonological Loop: The Digit Recall subtest requires children to listen to a sequence of digits and recall them. Because we did not want to confound STM performance with a general difficulty with mathematical information in our weaker maths group we also measured verbal STM using the Word Recall subtest. This subtest requires children to listen to a series of real words and to recall them.  In both verbal STM subtests the item is scored as correct if the child recalls the series in the correct order.

*Central executive:* Listening span. The Listening Span subtest requires children to listen to sentences and to decide whether the sentences are true or false. Children then recall the final word of the each sentence. Where two or more sentences occur in the same trial, the children must process the content of each sentence. At the end of the final sentence the child must recall the final words of each sentence in the same order as they were presented. An item is scored as being correctly processed if the child correctly decides whether the sentence is true or false. An item is scored as being correctly recalled if the child recalls the final words of the sentences in correct order.

Visual working memory

*Short-term memory (STM;* Visuo-spatial sketch pad): The Dot Matrix subtest requires children to view the position of a red dot in a series of four by four matrices and to repeat the sequence by tapping on the computer screen. An item is scored as correct if the child recalls the sequence in the correct order.

*Central executive:* Odd-one-out (OOO). In the OOO task three abstract shapes are presented on the computer screen. One of the shapes differs from the other two shapes and children must identify the odd shape by tapping it on computer screen. The shapes then disappear and the child must recall where the odd shape was located by tapping one of three empty boxes on the computer screen. Where two or more sets of shapes occur in the same trial, the children must identify the odd shape in the first set, then in the subsequent set(s). At the end of the final set of shapes, the shapes disappear and the children must recall the locations of the odd shapes in the same order as presented. An item is scored as being correctly processed if the child correctly identifies the odd shape. A set is scored as being correctly recalled if the child recalls the locations of the odd shapes in the correct order. A set is also scored as being correctly recalled if the child misidentifies the odd shape but correctly recalls the location of the misidentified shape during recall.

Standardized *recall* scores were measured for all subtests ('Digit Recall', 'Word Recall', 'Dot Matrix', 'List Recall Storage' and 'OOO Recall' in Results) and standardized *processing* scores were measured for OOO and Listening Recall ('OOO Processing' and 'List Recall Processing' in Results). Raw scores were also measured for OOO processing/ recall and Dot Matrix.

Mental rotation

Our mental rotation task was based on Quaiser-Pohl's Picture Rotation Test (Quaiser-Pohl, 2003). Three separate worksheets with different stimuli types (objects/animals, letters and hands) were presented to the children in a counterbalanced order; each worksheet had seven items.

Stimuli

The object/animal stimuli were obtained from:

<http://www.nefy.ucl.ac.be/facecatlab/stimuli.htm>

These stimuli are based on the widely used line drawings of Snodgrass and Vanderwart (1980). Object and animal pictures familiar to children, with no rotational or reflective symmetry were selected.

For the letter rotation task seven letters with no reflective or rotational symmetry were used (F, G, J, K, L, P, R,). Letters were presented in uppercase and in Arial font.

The stimulus set for the hand stimuli was kindly provided by Professor Lawrence Parsons (Parsons, 1994), which comprised of line drawings of left and right hands “viewed from five cardinal perspectives” (p. 711). The views of the different hands were front, back, and two other more unusual views e.g. side view for each hand (see Parsons, 1994, for stimuli).

For each item within a worksheet, a target stimulus was presented, along with three comparison stimuli, two of which are mirror images (distractors) and one was identical to the target. All three comparison images are rotated by various angles, and the child has to identify and circle the stimulus identical to the target. Rotation of the comparison stimuli was between 60 and 300 degrees (at intervals of 60 degrees resulting in five levels: 60, 120, 180, 240, 300). The position of the identical stimulus within the three comparison pictures was chosen at random, as was the angle of rotation at all three positions (under the condition that the same angle did not occur twice).

Procedure

The child was instructed to circle “the picture that is facing the same way when you imagine them all the right way up” and to complete all seven items on each page as quickly as possible. The children's total response time to complete all seven items was timed separately for the three pages.

Trail Making Task

The Trail Making Task (TMT) A and B were administered. In the TMT A the children were required to connect a series of numbered circles which were positioned across an A4 page. Children were instructed to connect the circles in sequence without removing their pencil from the paper, if possible, and to connect the circles as quickly as they could. The time to complete the sequence was recorded and the test was scored with 2 points if it was completed correctly or if self-corrected errors were made. A score of 1 was given for sequences that were mostly correct but for one uncorrected error and a score of 0 was given if the sequence was clearly incorrect or if the path could not be determined.

In the TMT B the children were presented with a similar page of numbered and lettered circles, and were required to connect the circles in sequence, however they were required to switch between numbers and letters (e.g., 1 - A - 2 - B - 3 - C and so on). The time to complete the sequence was recorded and the accuracy of the responses were scored using the same scoring scheme as for the TMT A.

Symmetry drawing

In the symmetry drawing task children were presented with two pages which contained six half drawn shapes against a grid background with a line of symmetry indicated with a dashed line. Children were required to draw the other half of the shape for each item. The shapes increased in difficulty from a simple rectangle to a series of steps. Shapes (and lines of symmetry) were presented vertically on one page and horizontally on the other. Children were instructed to complete the other half of the shape and if they found an item too difficult to move onto the next shape or the next page. Half of the children received the horizontally-oriented shapes first and the other half of the children received the vertically-oriented shapes first. The total time to complete the 12 shapes was recorded and the accuracy of items was scored with one point for every correct line segment.

Computerized Experimental Tasks

The following tasks were presented by the Presentation program of Neuro-behavioral Systems using a laptop computer. Unless described otherwise, reaction time and accuracy were recorded for all trials.

Simple RT

Children were instructed to press the space bar with their dominant hand as quickly as they could when a white box appeared on the screen. The white box, measuring 100 x 100 pixels, appeared at the centre of the screen for 3000 ms or until the child made a response. The delay before the square appeared was 1,000, 2,500 or 4,000 ms. The children received 6 practice trials before completing two blocks of 30 trials. For permutation testing DD minus control mean performance for all conditions was computed for accuracy and median RT. Additional ANOVAs and ANCOVAs were run with Group × Delay factors.

Sustained Attention

Children were required to attend to a stimuli stream and to detect a target sequence. Seven letters of the alphabet (from 'A' to 'H') were used. White letters on black background followed each other in rapid succession (300 ms). One letter was presented for 300 ms. The task was to continuously attend the stimuli stream and detect and indicate with a button press if the series of 'A B C' was seen, but not to respond if other combinations including A, B and C were presented, e.g. 'A C B' or 'A B D' etc.

There were three types of letter series:

1. targets (A B C)
2. 'deceivers'
   1. series beginning with A, then B followed by a letter out of the target range [D to H]
   2. series beginning with an A and ending with a C, but with another letter in the middle [D to H])
   3. series beginning with a letter D to H but ending with B and C
3. non-targets: random combinations of one letter from the target range [A B C] and two letters out of the target range [D to H]

The series were pseudo-randomized so that more than two target series could not follow each other, however, there was no break in between the letter 'triads'; letters were presented in one continuous stream.

There were 80 of each type of letter series (3 letter types x 80 triads x 3 letters in total).

Children completed a practice block of 24 triads, in which the letters in the target series were highlighted in red and underscored with yellow. These indicators served to familiarise the children with the target sequence during practice and were removed for the experimental block.

The number of hits and misses for targets, and the RT for target hits was recorded. Furthermore the number of correct rejections and false alarms for deceivers and non-target trials was recorded.

For permutation testing DD minus control mean performance was computed for accuracy and median RT.

Stop Signal Task

In the Stop Signal Task, a white arrow, pointing left or right, was shown for 500 ms on a black background in the middle of the screen. The arrow was either followed by a sound, the stop signal, or there was no sound. The time delay until the stop sound was dynamically varied between 0 and 1000 ms (see below). The ratio of 'go' and 'stop' trials was 2:1. Trials were separated by the presentation of an eye for 500 ms.

On 'go' trials, participants had to press the left 'Ctrl' button on the keyboard if the arrow pointed left and the right 'Ctrl' button if the arrow pointed to the right (hit: correct button press; miss: no button press). On 'stop' trials the stop signal was played after the arrow, indicating that participants should withhold their response to the arrow (correct rejection: no button press at stop signal; false alarm: button press when there was a stop signal). The stop signal sound was a 500Hz tone with a 10 ms rise- and fall time which played for 500 ms.

The time delay until the stop signal (sound) after the presentation of the arrow was set from trial to trial, depending on how the participants performed on the previous 'stop' trial. After an unsuccessful 'stop' trial (i.e. when they could not stop after the stop signal) the delay was decreased by 50 ms; after a successful 'stop' trial the delay was increased by 50 ms. Note that a shorter stop signal delay after the arrow makes the task easier because participants may not have begun to initiate their motor response yet so it is easier to inhibit, whereas a longer delay makes the task more difficult because the longer the delay the more likely the participant will have initiated their response.

Participants completed 12 practice trials before completing three experimental blocks of 60 trials. Stimuli series were randomized for each participant separately. For each trial we measured RT, sstRT (defined as the RT - average stop signal delay), and the number of times the child responded to the arrow incorrectly.

For permutation testing DD minus control mean performance on hits and correct rejections was computed for accuracy and median RT. Additional ANOVAs and ANCOVAs were run with Group × Condition (hit vs. correct rejection) factors.

Animal Stroop Task

This task was used by Szűcs et al. (2009b). Stimuli were coloured pictures of two animals presented in the middle of the computer screen. One animal was presented to the left, the other to the right of the centre. Subjects were instructed to press a button on the side corresponding to the animal which was bigger in real life. In the congruent condition the animal which was larger in real life was presented in a larger picture than the animal which was smaller in real life. In the incongruent condition the animal which was larger in real life was presented in a smaller picture than the animal which was smaller in real life. Subjects gave behavioural responses by pressing a button on a keypad with the left or right index finger. Stimulus series were carefully constructed so that consecutive stimuli could not induce response/processing bias. This was achieved by assuring that congruent and incongruent epochs requiring either a left or a right hand response were preceded by epochs belonging to each response hand and congruency condition an equal number of times.

Each trial consisted of a fixation sign (drawing of an eye) shown for 500 ms followed by a 1000 ms blank period, and the stimulus presented for a maximum of 3000 ms. The stimulus disappeared when the subject gave a response. The offset of the stimulus was followed by a 1000 ms blank period. There were 2 blocks of 48 stimuli. The experiment was preceded by 12 practice stimuli (half congruent).

Permutation testing compared the congruency effect (incongruent minus congruent condition) across groups. That is, DD minus control congruency effect was computed for accuracy and median RT. Additional ANOVAs and ANCOVAs were run with Group × Congruency factors.

Numerical Magnitude comparison Stroop task

This task was used by Soltész et al. (2011). Stimuli were pairs of Arabic digits shown simultaneously in the middle of the computer screen. Stimuli were white on black background. There were 4 possible number pairs, with two different numerical distances: distance 7: 1-8, 2-9, and distance 1: 1-2, 8-9. Children were instructed to decide which item of the pair was numerically larger than the other one. Children pressed a response button on a keypad where they detected the numerically larger stimulus. The correct response had to be given with the right hand in 50% of the cases, and with the left hand in the other 50%. Numerical and physical size information could be neutral, congruent or incongruent with each other in equal proportions. In the congruent condition the numerically larger (smaller) digit was also physically larger (smaller) than the other one (e.g., 1 8). In the incongruent condition the numerically larger (smaller) digit was physically smaller (larger) than the other one (e.g., 2 9). In the neutral condition both digits were of the same physical size (e.g., 1 2).

Each trial began with a fixation sign (a drawing of an eye) shown for 500 ms. After a 500 ms pause, a pair of stimuli were shown for maximum 3 s, or until the subject gave a response. The stimuli were followed by a pause of 500 ms. The viewing distance was approximately 60 cm. Physically small stimuli had a font-size of 40, physically large stimuli had a font-size of 50. Stimulus pairs with one small and one large-sized stimulus subtended a view angle of 2.4° horizontally, and 1° vertically. In half of the neutral trials both digits had a font size of 40 (horizontal view angle: 1.9°; vertical view angle: 0.7°), in the other half both digits had a font size of 50 (horizontal view angle: 2.4°; vertical view angle: 1°). 96 trials were presented in 2 blocks. The experiment was preceded by 12 practice trials.

Permutation testing compared the distance effect (distance 7 minus distance 1), facilitation effect (congruent minus neutral) and interference effect (incongruent minus neutral) across groups. That is, DD minus control effects were computed for accuracy and median RT. Additional ANOVAs and ANCOVAs were run with Group × Congruency (neutral, congruent, incongruent) × Numerical Distance (1 vs. 7) factors.

Physical size comparison Stroop task

This task was used by Szűcs et al. (2009a). The physical size decision Stroop task was identical to the numerical magnitude Stroop task, with the exception that the children were required to respond to the physically larger stimulus. Congruent and incongruent trials were exactly the same as for the numerical magnitude decision task, however in neutral trials the digits differed in physical size but were numerically identical (e.g., 1 1).

Permutation testing compared the distance effect (distance 7 minus distance 1), facilitation effect (congruent minus neutral) and interference effect (incongruent minus neutral) across groups. That is, DD minus control effects were computed for accuracy and median RT. Additional ANOVAs and ANCOVAs were run with Group × Congruency (neutral, congruent, incongruent) × Numerical Distance (1 vs. 7) factors.

Subitizing

An array of dots appeared on the screen and the children were instructed to say the number of dots as quickly as possible. The number of dots in each array ranged between one and six. For each set size, an array was constructed using dots which were one of five sizes; the diameter of each dot within an array was 5, 7.5, 10, 12.5 or 15 mm. The dot stimuli were black, were presented in canonical and, where possible, non-canonical arrangements, in the centre of the screen against a white background.

Children's reaction times were measured using a voice key. After the children responded the experimenter also entered the child's answer using an external numerical keyboard, which was necessary for scoring the accuracy of responses, and advanced the experiment to the next trial. Children completed six practice trials (one of each set size) before completing two blocks of 30 trials (each block contained all combinations of the six set sizes and five dot sizes).

Permutation testing compared the slope of decision curves by group in the subitizing range (numbers 1-3) and in the counting range (numbers 4-6). That is, DD minus control slopes were computed for both ranges for accuracy and median RT. Additional ANOVAs and ANCOVAs were run with Group × Number range (subitizing [1-3] vs. counting [4-6]) × Number (1, 2 and 3; or 4 5 and 6) factors.

Symbolic Magnitude Comparison task (Distance Effect)

This task was similar to the magnitude comparison task used by Soltész and Szűcs (2009). In the symbolic magnitude comparison task participants decided whether visually presented digits (1, 4, 6 or 9) were smaller or larger than 5. White digits with a font-size of 40 were presented in the middle the computer screen on a black background. Trials started with a picture of an eye shown for 200 ms. After 1000 ms a digit was shown for 3000 ms or until the child made a response. 400 ms passed before the next eye appeared. Children pressed a button on the keyboard with their left hand if the number was smaller than 5 and another button with their right hand if the number was larger than 5. Two blocks of 40 stimuli were presented, preceded by 8 practice stimuli before the first block.

Permutation testing compared the slope of decision curves in both groups. That is, DD minus control slopes were computed for accuracy and median RT. Additional ANOVAs and ANCOVAs were run with Group × Distance (1 vs. 4) factors.

Nonsymbolic magnitude comparison task (non-symbolic ratio effect)

This task was used by Soltész et al. (2010). Black dots on white background were used as stimuli. Two sets of dots were presented simultaneously on the computer screen. The sets were separated by 7.5 cm, and were visually easily distinguishable from each other. The overall envelope (corresponding to contour length in Rouselle et al., (2004)) of a set was kept constant at 9×9 cm, as overall envelope has been found to help children even in conditions where overall surface is incongruent with number (Barth et al., 2005). Children’s task was to find out which set contains more dots and press the button on the side of the larger set. Response side was counterbalanced.

The size of dots was constant within set and varied between sets. The individual size of dots and the pattern of dots were randomly varied through pairs of sets. Sets with the same number of items never had the same dot size. Only numerosities above the subitization range were used in order to exclude that object-based attention (or the object file system, Simon, 1997; Kahneman, Treisman & Gibbs, 1992; Uller et al., 1999; Huttenlocher et al., 1994) would be used to complete the comparison task.

The following factors were taken into consideration: (1) The ratio of the number of dots in the two sets. (2) The numerical distance between the number of dot in the two sets. (3) The type of the physical control variable. (4) The congruity of physical control variables and numerosity. (5) The overall numerical sum of items in a display. The ratios and numerical distances for all combinations of numerosities are summarized in Supplementary Methods Table 1. Accordingly, all number pairs used are shown in Table 1. There was no extreme large numerical distance for the 2:3 ratio because the sum of items would have been much larger than in other conditions (at least 16:24, sum is 40; or 20:30, sum is 50). Rather, we decided to keep the sum of items for the largest numerical distances in the 1:2 and 3:5 ratio conditions to be approximately equal to the sum of items in the distance 6 condition in the 2:3 ratio condition. This allowed for checking for ratio effects independent from the overall sum of items in the display.

Two different physical variables were manipulated as controls: the overall surface (hence, luminance) and the overall circumference (sum of the individual items’ circumferences) of the dot groups. These two physical controls were intermixed during stimulus presentation. The ratios of the overall physical sizes (surface in half of the trials and circumference in other half of the trials) of dot sets were congruent or incongruent with the numerical ratio of the dot sets. In the congruent condition the more numerous set was larger in overall physical size than the less numerous set. In the incongruent condition the more numerous set was smaller in overall physical size than the less numerous set. Congruent and incongruent trials were pseudo-randomly intermixed (no more than three of each could follow in a sequence).

In each trial the ratio of perceptual features of the two dot patterns was kept the same as their numerical ratio. This was done because if the ratio of the surfaces or circumferences within a set pair were not in accord with the ratio between the numbers in the set pair, the influence of perceptual variables would differ among numerical ratios. For example, if the ratio between the numbers were 1:2 and the ratio between perceptual variables were 2:3, the perceptual difference would be less salient than the numerical difference. This would result in better numerical discrimination performance solely because physical variables would be less distractive. Similarly, if the perceptual ratio were 1:2 and the numerical ratio were 2:3, the perceptual difference would be more salient. Our design avoided this problem.

In order to investigate the effects of numerical distance and ratio independent from each other, separate analyses were performed on number pairs varying in numerical distance while ratio was kept constant, and on number pairs with identical numerical distances and with varying ratio.

Each trial consisted of a fixation sign (brackets) shown for 500 ms followed by a 1000 ms blank period, and the stimulus stayed on the screen until the subject gave a response. The offset of the stimulus was followed by a 1000 ms blank period. There were 4 blocks of 30 stimuli. The experiment was preceded by 12 practice stimuli.

Permutation testing compared the slope of decision curves and the size of the congruency effect in both groups. That is, DD minus control slopes and DD minus control congruency effects were computed for accuracy and median RT. Additional ANOVAs and ANCOVAs were run with Group × Congruency (congruent vs. incongruent) × Ratio (1:2, 3:5 and 2:3) factors.

**Supplementary tables**

Table 1: The dot number pairs per each ratio.

| **1 : 2** | **3 : 5** | **2 : 3** |
| --- | --- | --- |
| 4 8 * | 6 10 * | 8 12 * |
| 6 12 ** | 9 15 ** | 12 18 **/° |
| 10 20 ° | 12 20 ° |  |

Table from Soltész et al. (2010). Ratios are in columns. * and ** indicate dot pairs which are the same numerical distance (the numerical distances are 4 and 6, respectively). The overall sum is almost equal for dot number pairs marked with ° (30, 32 and 30)

**Supplementary References**

Alloway TP. Automated Working Memory Assessment (AWMA). London: UK: Pearson Assessment; 2007.

Baddeley AD, Hitch GJ. Working memory. In: Bower GA, Editor. The Psychology of Learning and Motivation. New York: Academic Press; 1974. p. 47–89

Barth H, La Mont K, Lipton J. Spelke, ES. Abstract number and arithmetic in preschool children. Proc Natl Acad Sci U S A 2005; 102(39): 14116-14121.

Huttenlocher J, Jordan N, Levine SC. A mental model for early arithmetic. J Exp Psychol Gen 1994; 123: 284-296.

Kahneman D, Treisman A, Gibbs BJ. The reviewing of object files: object-specific integration of information. Cog Psychol 1992; 24: 175-219.

Parsons L. Temporal and kinematic properties of motor behavior reflected in mentally simulated action. J Exp Psychol Hum Percept Perform 1994; 20: 709-730.

Quaiser-Pohl C. The mental cutting test “Schnitte” and the picture rotation test-two new measures to assess spatial ability. International Journal of Testing 2003; 3: 219-231.

Raven J. Raven’s - Educational: Coloured Progressive Matrices (CPM). London: Pearson Assessment; 2008.

Rousselle L, Palmers E, Noël M-P. Magnitude comparison in preschoolers: What counts? Influence of perceptual variables. J Exp Child Psychol 2004; 87: 57-84.

Sattler J. Assessment of Children, 3rd Edition. San Diego: Jerome Sattler; 1992.

Simon TJ. Reconceptualizing the origins of number knowledge: A non-numerical account. Cogn Dev 1997; 12: 349-372.

Snodgrass J, Vanderwart M. A standardized set of 260 pictures: Norms for name agreement, image agreement, familiarity, and visual complexity. J Exp Psychol Hum Learn 1980; 6: 174–215. doi:10.1037/0278-7393.6.2.174

Soltész F, Szűcs D. An electro-physiological temporal principal component analysis of processing stages of number comparison in developmental dyscalculia. Cogn Dev 2009; 24(4): 473–485. doi:10.1016/j.cogdev.2009.09.002

Soltész F, Szűcs D, Szűcs L. Relationships between magnitude representation, counting and memory in 4- to 7-year-old children: a developmental study. Behav Brain Funct 2010; 6. doi:10.1186/1744-9081-6-13.

Soltész F, Goswami U, White S, Szűcs D. Executive function effects and numerical development in children: Behavioural and ERP evidence from a numerical Stroop paradigm. Learn Individ Differ 2011; 21(6): 662–671. doi:10.1016/j.lindif.2010.10.004

Szűcs D, Soltész F. White S. Motor conflict in Stroop tasks: direct evidence from single-trial electro-myography and electro-encephalography. Neuroimage 2009a; 47(4): 1960–73. doi:10.1016/j.neuroimage.2009.05.048.

Szűcs D, Soltész F, Bryce D, Whitebread D. Real-time tracking of motor response activation and response competition in a Stroop task in young children: A lateralized readiness potential study. J Cogn Neurosci 2009b; 21(11): 2195–206. doi:10.1162/jocn.2009.21220

Uller CM, Carey S, Huntley-Fenner G, Klatt L. What representations might underlie infant numerical knowledge. Cogn Dev 1999; 14: 1-36.

Vincent D, Crumpler M. Hodder Group Reading Tests 1-3 (II.). London: UK: Hodder; Education; 2007.

Wechsler D. Wechsler Intelligence Scale for Children -Third Edition (WISC-III). San Antonio, TX: Psychological Corporation; 1991.

Wechsler D. Wechsler Individual Achievement Test - Second UK Edition (WIAT-II UK). London: UK: Harcourt Assessment; 2005.

Williams J. Mathematics Assessment for Learning and Teaching. London: UK: Hodder Education; 2005.
